# BlephEx-treatment for blepharitis: a prospective randomized placebo-controlled trial

**DOI:** 10.1186/s12886-024-03765-3

**Published:** 2024-11-18

**Authors:** Helena Siegel, Annika Merz, Nikolai Gross, Marie-Christine Bründer, Daniel Böhringer, Thomas Reinhard, Philip Maier

**Affiliations:** 1 Eye Center, Medical Center – University of Freiburg, Faculty of Medicine, University of Freiburg, Killianstraße 5, Freiburg, 79106 Germany; 2 Ophthalmological Practice, Leopoldring 5, Freiburg, 79098 Germany; 3 Department of Ophthalmology, University Medicine Greifswald, Ferdinand-Sauerbruch-Straße, Greifswald, 17475 Germany

**Keywords:** Eyelid margin care, BlephEx™ device, Blepharitis, Ocular surface, Meibomian gland, Tear film

## Abstract

**Background:**

Blepharitis is a chronic inflammatory condition of the eyelids that affects a large proportion of patients in eye care settings. First-line treatments provide only partial relief for many patients. The BlephEx™ device provides automated eyelid debridement and aims to remove pathogenic biofilms from the eyelid margin to treat blepharitis long-term. However, evidence supporting the efficacy of BlephEx™ is limited.

**Methods:**

In this double-masked randomized controlled trial, 42 patients with symptomatic blepharitis refractory to treatment were assigned to the BlephEx™ treatment or sham treatment group. Outcome measures including Ocular surface disease index (OSDI), tear break-up time (TBUT), Schirmer test, and Efron grading scale scores were assessed at baseline and after 4 weeks. A crossover design in which the treatment groups were swapped after 4 weeks was used as a recruitment tool. After receiving treatment, two patients (one per group) were lost to follow-up.

**Results:**

The sham group exhibited a significant decrease in the Efron Grading Scale score. No significant differences were observed in the other outcomes between the two groups. The BlephEx™ group showed slightly greater decreases in the OSDI and Efron grading scale scores and an increase in the TBUT than did the sham group, but these differences were not statistically significant. Mild discomfort was the most common side effect and occurred equally in both groups.

**Conclusions:**

No significant difference in outcomes was observed between patients who underwent BlephEx™ therapy and those who received sham treatment. BlephEx™ treatment cannot be recommended for treating blepharitis.

**Trial registration:**

Retrospectively registered on February 16, 2024 in the DRKS (German Clinical Trials Register under https://drks.de/search/de/trial/DRKS00033492) under the trial registration number DRKS00033492.

## Introduction

Blepharitis is a chronic inflammatory condition of the eyelids that can lead to dry eye disease and irritation. It affects up to 47% of patients in eye care settings [1–3]. While first-line treatments such as warm compresses, eyelid massage, and lubricants provide relief for some patients, many patients continue to have persistent inflammation and symptoms that significantly impact quality of life [4–6]. More intensive treatments, such as topical cyclosporine and other topical immunomodulatory drugs, require high patient compliance and proper techniques to be effective [7, 8].

**Fig. 1:**
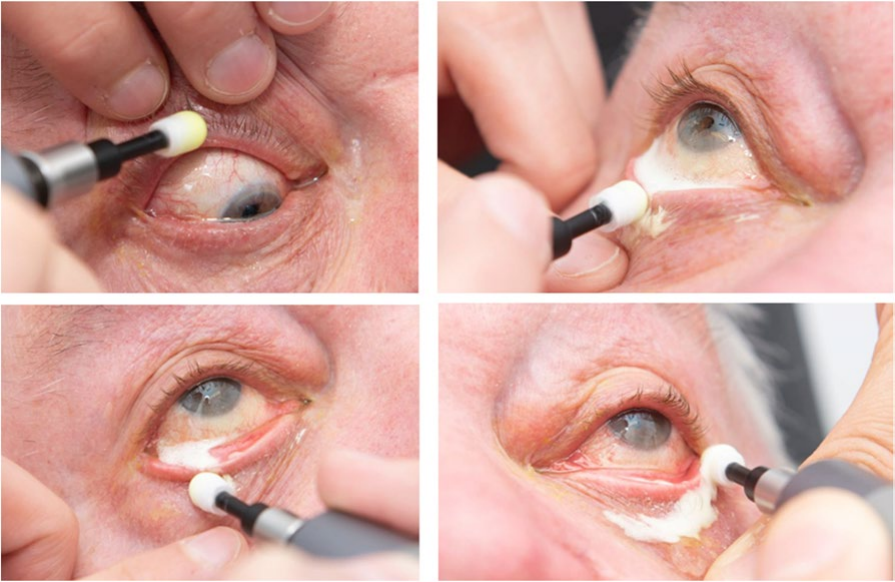
Photographic guide for BlephEx™ treatment: The cleansing head was placed on the BlephEx™ device, and a cleansing gel was applied. Then, the edges of the upper and lower eyelids are treated

For patients with refractory blepharitis, new treatment options that mitigate the limitations of current therapies are needed [3, 9–11]. The BlephEx™ (Scope Ophthalmics, London, UK) device provides automated eyelid margin debridement and has been proposed as an alternative treatment for blepharitis. BlephEx™ aims to disrupt pathogenic biofilms on the eyelid margin that contribute to chronic inflammation and symptoms [12]. It can be used to remove encrusted secretions, patches, flakes, etc., on the eyelid margins and in the excretory ducts of the meibomian glands (see Fig. 1) so that pathogenic biofilms can be removed in the long term. In the case of chronic blepharitis, treatment should be repeated every four to six months according to the manufacturer’s instructions [12]. However, evidence for the efficacy of BlephEx™ remains limited, with few controlled studies published to date.

## Materials and methods

### Trial design

The aim of this randomized, placebo-controlled, double-blind, prospective trial was to examine the efficacy of BlephEx™ treatment for chronic blepharitis by determining whether BlephEx™ treatment improves quality of life and ocular surface parameters in patients with chronic blepharitis compared to sham treatment. Our primary outcome was the change in the Ocular Surface Disease Index (OSDI), a validated metric for quality of life related to dry eye disease and blepharitis. Secondary outcomes included the severity of eyelid inflammation measured by the Efron grading scale for eyelid margin disease, tear break-up time (TBUT), and Schirmer's test to evaluate tear production.

This study aimed to assess whether automated eyelid margin debridement with BlephEx™ provides benefits beyond a sham procedure, which controls for mechanical eyelid stimulation and placebo effects. Patients were randomized to receive either BlephEx™ debridement per the manufacturer’s instructions or sham treatment under equivalent conditions. Outcomes in the BlephEx™ group were compared to those in the sham group to determine the impact of BlephEx™ treatment relative to stimulation and placebo effects alone.

**Fig. 2:**
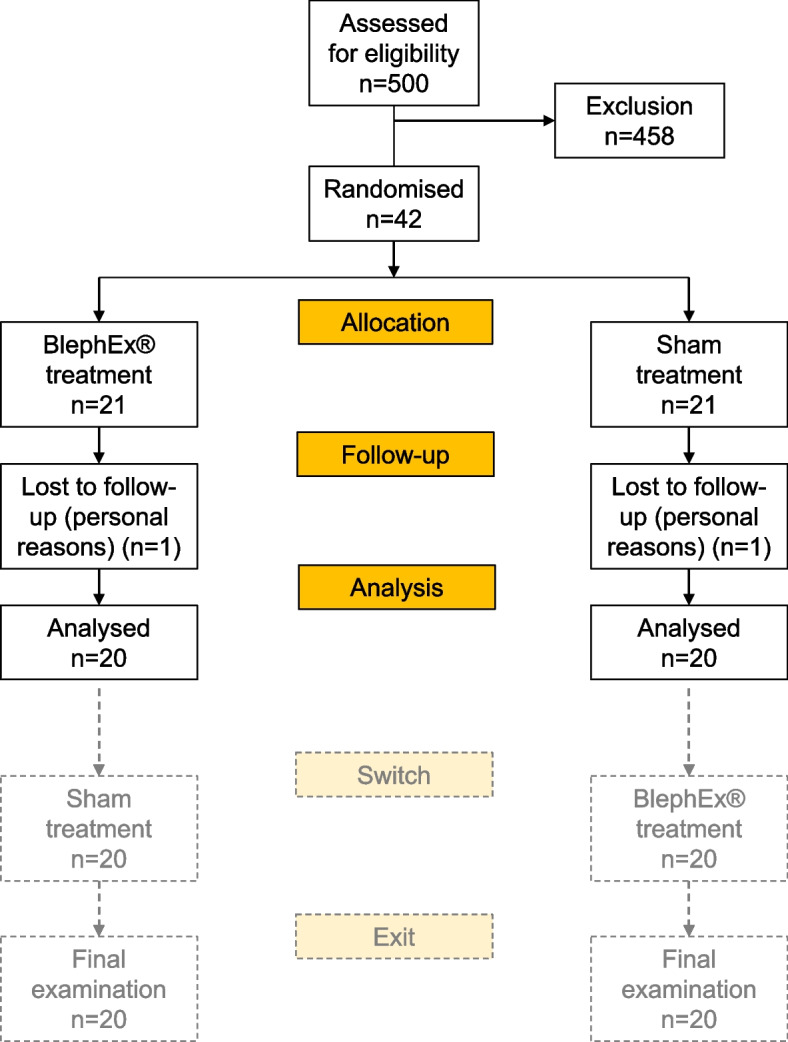
Patient flowchart study: study enrollment and progression

The study was conducted at the Eye Center of the University Hospital Freiburg after approval by the local ethics committee (application no. EK-Freiburg 349/17) and can be accessed in the research database of the University of Freiburg. (https://forschdb.verwaltung.uni-freiburg.de/forschdbukl/rech_frame_projekt.htm as of 02.01.2023). The study conformed to the Declaration of Helsinki. The trial registration was carried out retrospectively on February 16, 2024 in the DRKS (German Clinical Trials Register) at https://drks.de/search/de/trial/DRKS00033492) under the trial registration number DRKS00033492. A crossover design was used as a recruitment aid (study participants received verum treatment in each case) and was not considered for the statistical analysis (see Fig. 2). Therefore, only the baseline (examination before treatment) and the examination 4 weeks after treatment (switch at the time of crossover) visits were evaluated to minimize pretreatment bias. The patients were divided into 2 groups randomized to receive either BlephEx™ or sham treatment. Afterwards, the participants were switched to the alternative intervention. The observed treatment effect in this second stage was not taken into account in the statistical analysis to avoid a potential carryover effect, which could have influenced the results.

### Trial participants

The inclusion criteria were symptomatic blepharitis (at least Grade 2 according to the Efron grading scale), age older than 18 years, decimal visual acuity > 0.5, blepharitis refractory to eyelid margin care for at least 4 weeks, and the use of lubricating drops.

The exclusion criteria were routine (daily) contact lens wear, other eyelid or ocular surface diseases, previous ocular surgery, previous treatment with cyclosporin A or steroids, pregnancy, autoimmune diseases (e.g., rheumatologic conjunctivitis, Sjögren's syndrome, endocrine orbitopathy) or other immunological diseases and acute infections. Further exclusion criteria were the presence of trachoma, atopic dermatitis, diabetes mellitus, severe systemic diseases, graft-versus-host disease or the use of antibiotics in the last 4 weeks.

The screening process was as follows: At the first contact in our outpatient clinic, patients with blepharitis were screened by the examining doctors according to our inclusion and exclusion criteria. If someone was generally eligible for participation in the study, a member of the study team was contacted. A special medical history form was used to assess all the inclusion and exclusion criteria. If all the criteria for participation in the study were fulfilled, the patients were informed and given a consent form. Subsequently, an appointment was made for the initial examination and treatment. Patients were recruited between December 2017 and June 2018.

In the case of a new occurrence of the disease, basic therapy, which included four weeks of eyelid margin care and lubricating drops, was recommended first. If the symptoms did not subside sufficiently within 4 weeks of treatment, the patient was included in the study.

### Interventions

BlephEx™ (Scope Ophthalmics, London, UK) treatment was performed according to the manufacturer’s instructions after topical anesthesia. The cleaning head was placed on the BlephEx™ device (one per eye), and Blephagel® from Théa Pharma GmbH was applied as a cleaning gel (composition: agua, poloxamer 188, PEG-90, carbomer, and sodium hydroxide). Then, the upper and lower lids of each eye were treated for two minutes according to the manufacturer's instructions. The practitioner moved along the eyelid margin and removed both coarse and minor "debris" (see Fig. 1).

The sham treatment was carried out under the same temporal and spatial conditions. The cleaning head was placed on the BlephEx™ device (one per eye), and cleaning gel was applied. Then, each eye was treated for two minutes on the upper and lower lid. However, the doctor did not move along the eyelid margin but rather along the adjacent row of eyelashes on the eyelid skin so that no mechanical cleaning of the eyelid margin could be assumed. Due to the anatomical proximity, the resulting transfer of sensation to the eyelid and the previous superficial anesthesia, it can be assumed that it was not possible for the patient to distinguish between BlephEx™ treatment and sham treatment.

### Outcome measures

The primary endpoint was a change in quality of life measured by the ocular surface disease index (OSDI), as described by Schiffman et al. [13]. The scores at the different time points were compared with each other and statistically analyzed at the end of the study.

The secondary endpoints were the severity of lid margin inflammation, graded according to the Efron grading scale, as well as the evaluation of tear break-up time (TBUT) and the Schirmer test.

The Efron grading scale was used to assess eyelid margin involvement in a blinded manner based on the eyelid margin photos before and 4 weeks after treatment; the assessments were performed by two independent blinded examiners on a scale of 0 to 4. The decisive factors were redness of the eyelid margin, condition of the meibomian gland excretory ducts, incrustations and adhesions on the eyelashes, telangiectasia on the eyelid margin, and visible surrounding skin irritations [14].

The tear break-up time (TBUT) was measured using a 2% fluorescein solution. Fifty microliters of this solution was dropped into the lower fornix with a pipette and distributed over the surface of the eye by blinking. This was followed by examination via a slit lamp in the photo laboratory with blue light. After a short exposure time, the patient was asked to close his eyes, open them on command and then keep them open. This was ensured by the examiner with the help of a cotton swab. The time until the tear film broke up was determined and recorded. The procedure was repeated three times for each eye.

The Schirmer's test II was performed to obtain information on baseline tear production. For this purpose, oxybuprocaine 0.4% eye drops were given, and after an exposure time of five minutes, the Schirmer test strips were hooked on the lateral lower lid. The patients were asked to close their eyes loosely. After 10 min, the test strips were removed, and a ruler was used to directly measure how much fluid the strips had absorbed.

Best-corrected visual acuity was assessed using Early Treatment of Diabetic Retinopathy Study (ETDRS) charts after standardized subjective refraction by trained personnel [15]. VA was converted to logMAR for statistical analysis.

### Sample size

Given that this was an exploratory approach, with no firm prior knowledge of effect sizes or expected variances, it was not possible to calculate the sample size beforehand.

### Randomization

#### Sequence generation

The randomization sequence was generated in advance with the package "blockrand" from the R-platform at a ratio of 1:1 for the verum patients to the control patients and was stored covertly for the study personnel in the database.

#### Allocation concealment mechanism and implementation

Randomization was automated upon study inclusion. The group allocation in combination with a randomization number was directly transmitted electronically to the treating physician only by e-mail. Moreover, it was ensured that this information was not visible to anyone involved in the diagnostic process during the course of the study.

### Blinding

The study treatments were performed by an unmasked study team of two ophthalmologists. Diagnostics were performed by a doctoral fellow, who was blinded to the treatment performed until the end of the study. The study team (two ophthalmologists) involved in the assessment of the Efron grading scale was also blinded at all times. All study patients were also blinded regarding the type of treatment (see description of sham treatment above).

### Statistical methods

Categorical data are presented as percentages. For the analysis of categorical variables, the Chi-Square Test was applied when the expected frequencies in all categories were above 5. In cases where the expected frequencies in any category were below 5, the Fisher's Exact Test was used to ensure the accuracy of the results. This approach ensures appropriate testing for both large and small sample sizes, depending on the distribution of the categorical data across groups.

All continuous data were tested for normality using the Shapiro–Wilk test. For normally distributed data (e.g., OSDI), the paired t-test was used to calculate the *p*-value of the change before and after treatment within each treatment group, and the unpaired t-test was used to calculate the *p*-value for differences between the two treatment groups.

For data that were not normally distributed, the Wilcoxon signed-rank test was applied to calculate the *p*-value for the change before and after treatment within a treatment group. The Mann–Whitney U test (also known as the Wilcoxon rank-sum test) was used to calculate the *p*-value for comparing differences between the treatment groups.

For each primary and secondary outcome, results for each group were reported, and the estimated effect size and its precision (95% confidence interval) were calculated in accordance with the Consolidated Standards of Reporting Trials (CONSORT) 2010 checklist. The difference before and after treatment was determined by calculating the mean and 95% confidence intervals, as well as the median and interquartile range (IQR). For normally distributed data, Cohen’s d was calculated to compare the effectiveness of the different treatments. Cohen proposed treating d = 0.2 as a "small" effect size, 0.5 as a "medium" effect size and 0.8 as a "large" effect size. This implies that if the difference between the means of two groups is less than 0.2 standard deviations, the difference is considered to be negligible, even if it is statistically significant.

For non-normally distributed data, the Rank-Biserial Correlation (rbc) was used as the effect size for comparisons made with the Mann–Whitney U test. While there are no universally agreed-upon thresholds for small, medium, or large effect sizes in the context of rbc, a similar framework to that of Cohen’s d was applied, with rbc ≈ 0.10 representing a small effect size, rbc ≈ 0.30 representing a medium effect size, and rbc ≈ 0.50 or higher representing a large effect size.

All statistical analyses were performed using R version 4.3.1 and 4.4.1 (R Foundation for Statistical Computing, Vienna, Austria).

## Results

### Participant flow

In this study, a total of 42 patients who met the inclusion criteria were recruited. Of these, two patients left the study prematurely (one per study arm). As a result, the study ended with 20 participants in each group (see Fig. 2).

### Baseline data

A detailed description of the baseline data can be found in Table 1.

**Table 1:** baseline demographic and clinical characteristics for each group at inclusion

	N	BlephEx	Sham
Age at inclusion [years]	42	47.2/57.7/76.1	46.8/74.1/78.5
Female	24	52% (11)	62% (13)
Postmenopausal hormone replacement therapy	18	33% (7)	52% (11)
Antidepressants	3	0% (0)	14% (3)
Oral beta blocker	8	19% (4)	19% (4)
Oral contraception	0	0% (0)	0% (0)
Conjunctivitis	0	0% (0)	0% (0)
Contact lens wear during the study	2	0% (0)	10% (2) *
Number of medications taken	42	0.0 /1.0 /3.0	0.0 /1.0 /3.0
BCVA (OD) [logMAR]	42	-0.1 / -0.1 / 0.1	-0.1 / 0 / 0.1
BCVA (OS) [logMAR]	42	-0.1 / -0.1 / 0	-0.1 / -0.1 / 0
Nicotine consumption	18	38% (8)	48% (10)
Pack years	18	2.75 / 11.0 /16.25	1.0 / 9.0 / 14.25
Efron grading at inclusion	42	2.0 / 3.0 / 3.0	2.0 / 2.5 / 3.0
OSDI** [points]	42	10.4 / 15.6 / 28.7	18.8 / 33.3 / 41.7
TBUT [s]	42	1.73/2.19/3.07	2.05/2.56/3.43
Schirmer's test II [mm]	41	5.75/11.50/21.12	7.50/11.50/13.00

Continuous data are presented as lower, median and upper quartile and categorical data are presented as percentage of all the patients of the respective group (absolute number in parentheses)

*BCVA* designates the best-corrected visual acuity in logMAR annotation, *OSDI* Ocular Surface Disease Index, *TBUT* Tear Break-Up Time, *OD* right eye, *OS* left eye

^*^Only sporadic wearing of contact lenses to exercise reported during the study period

^**^Statistically significant difference of the two groups (*p* = 0.020)

Except for the OSDI (Ocular surface disease index), no statistically significant difference was observed between the groups.

There was no statistically significant difference between the two study groups for any of the parameters shown, with the exception of the OSDI; here, despite randomization, there was a statistically significant difference between the two groups (*p* = 0.020), with a higher score in the sham group.

### Outcomes and estimation

The sham group showed a statistically significant decrease in the Efron grade after treatment (*p* < 0.05; see the detailed report in Table 2). No significant differences were observed in the other parameters before or after treatment in either group.

**Table 2:** Descriptive statistics and effect sizes for outcome measures in the BlephEx® and sham treatment groups

	Group			
	**BlephEx® treatment** **(*n* = 20)**	**Sham treatment** **(*n* = 20)**	***p*-value of the difference**	**Effect size**
**OSDI**				
*Mean (95%CI)*	-2.40 (-7.95 to 3.16)	-4.90 (-11.51 to 1.72)	0.548	0.19 (Cohen`s d)
*Median (IQR)*	-2.09 (-7.29/3.12)	-5.21 (-13.54/1.04)		
*p-value*	0.378	0.138		
**Efron grading**				
*Mean (95%CI)*	-0.2 (-0.56 to 0.16)	-0.3 (-0.57 to -0.03)	0.730	-0.095 (rbc)
*Median (IQR)*	0 (-1.0/0)	0 (-1.0/0)		
*p-value*	0.276	0.041		
**TBUT [s]**				
*Mean (95%CI)*	0.81 (-0.9 to 2.52)	-0.20 (-1.21 to 0.81)	0.277	-0.205 (rbc)
*Median (IQR)*	0.22 (-0.13/0.76)	-0.01 (-0.55/0.59)		
*p-value*	0.240	0.808		
**Schirmer [mm]**				
*Mean (95%CI)*	0.94 (-1.74 to 3.61)	0.95 (-1.05 to 2.95)	0.787	-0.053 (rbc)
*Median (IQR)*	2.00 (-3.63/3.75)	1.00 (-1.25/2.13)		
*p-value*	0.717	0.397		

This table shows the mean values and 95% confidence intervals (95%CI, as well as the median values and interquartile ranges (IQR). The columns further include the treatment group designation (group) and the effect size calculated calculated for between-group comparisons. For the OSDI, where the data were normally distributed, Cohen's d was used to calculate the effect size, and the paired t-test was used to calculate the *p*/value of the change before and after treatment, and the unpaired t-test was used to calculate the p-value comparing the difference between the treatment groups. For all other outcome measures, which were not normally distributed, the Wilcoxon signed-rank test was used for within-group comparisons, and the Mann-Whitney U test was applied for between-group comparisons. In these cases, the Rank-Biserial Correlation (rbc) was used to report effect sizes, as it is more appropriate for non-parametric data.The outcome measures were the OSDI (Ocular Surface Disease Index), the Efron grading scale, the TBUT (Tear Break-Up Time) und Schirmer II test results.

When the BlephEx™ treatment and the sham treatment were compared, the OSDI score, Efron grade, and TBUT decreased slightly more in the BlephEx™ group, whereas the Schirmer test increased. However, the effect sizes showed that the OSDI score and Efron grade had negligible effects, while the TBUT had only a minor effect. Furthermore, none of the differences in outcome measures (OSDI score, Efron grade, TBUT, or Schirmer test score) between the treatment groups were statistically significant, as demonstrated by *p* values greater than 0.05.

### Safety

Visual acuity was collected as a safety measure and showed no significant decrease in either group.

Immediately after the treatment, three patients in the BlephEx™ group and two patients in the sham group reported eye irritation for a couple of hours. One patient in the BlephEx™ group reported having unilateral itching that persisted up until follow-up and started one week after therapy. One patient in the Sham group disclosed having epiphora, which had only started upon receiving treatment.

## Discussion

The purpose of this investigator-initiated, double-masked, randomized controlled trial was to determine the effectiveness of eyelid margin care utilizing the BlephEx™ device in minimizing blepharitis-related complaints. Although a small difference favoring the BlephEx® treatment group was observed for the TBUT, this difference was not statistically significant compared the sham treatment group, and the negligible effect size indicate that this difference is unlikely to be clinically meaningful. Interestingly, the sham group showed a greater reduction in OSDI scores (-4.90) compared to the treatment group (-2.40), suggesting that the mechanical stimulation involved in the sham procedure may have contributed to the placebo effect observed in this study. While there was no significant difference between the groups, the reduction in symptoms in the sham group highlights the potential impact of non-specific therapeutic interventions, which has also been noted in prior studies. Mechanical stimulation of the eyelid margin, even in the absence of active exfoliation, could increase tear production or improve tear film distribution, leading to symptomatic relief. This aligns with the significant decrease in the Efron grading scale score in the sham group, indicating a reduction in blepharitis severity. It is crucial to note, however, that this improvement was not detected in the other measures, such as the OSDI score, TBUT, or Schirmer test results.

Different types of eyelid exfoliation techniques have been shown to produce varying effects on dry eye disease [16]. A study by Siddireddy et al. [17] demonstrated that micro-sponge exfoliation primarily improves microorganism load and lipase activity, whereas others studies found diamond bur exfoliation to enhance meibomian gland secretion [18–20]. This variability suggests that BlephEx™, which uses a micro-sponge, may have a more pronounced impact on reducing bacterial load rather than directly improving meibomian gland function.

A recent systematic review by Ballesteros-Sánchez et al. [16] summarizes a part of these findings and supports the potential benefits of eyelid exfoliation in managing dry eye disease and blepharitis, with improvements in OSDI and TBUT. Nonetheless, the reduction in microorganism load reported in the review is relevant for the management of blepharitis and aligns with our study’s objectives. Another interesting finding in this review was that control groups receiving basic eyelid hygiene also showed significant improvements in OSDI, highlighting the potential placebo effect of mechanical stimulation. This aligns with our findings, where the sham group showed a greater reduction in OSDI scores than the treatment group, despite no significant difference between them.

Based on our results, however, the BlephEx™ as a method for eyelid exfoliation does not appear to provide significant added benefit over sham treatment for the general management of chronic blepharitis. The small sample size is a limitation, but the lack of meaningful effects suggests limited utility for widespread adoption of this specialized equipment. Patients could achieve similar placebo effects from simpler, cost-effective in-office eyelid hygiene techniques. These results are in line with earlier research that found conflicting evidence for the effectiveness of mechanical eyelid margin debridement in the treatment of blepharitis.

To date, only a few other studies have specifically examined the effect of BlephEx™ treatment in chronic blepharitis, and even fewer have been placebo controlled. One of the studies without a control group was conducted by Epitropoulos et al. [21] and involved 177 patients from various centers who underwent BlephEx™ treatment in a retrospective data analysis. In this study, all the measurements (TBUT, SPEED score, and OSDI score) indicated a significant improvement following BlephEx™ treatment. Mulder et al. [22] reported significant improvements in the TBUT, OSDI, and Efron grading scale scores after BlephEx™ treatment in a prospective study of 20 patients. However, both of these studies lacked a control group and were never published in a scientific journal. In another retrospective case series study of 24 patients with moderate to severe meibomian gland dysfunction, Moon et al. [23] reported that eyelid debridement using BlephEx™ in combination with eyelid margin care improved clinical findings, subjective symptoms, meibomian gland function, and ocular surface MMP-9 levels. However, since no control group was included in this study either, no clear conclusion can be drawn on the real treatment effect compared to a possible placebo effect.

Recent studies, such as those by Siddireddy et al. [17, 24], further explore the effects of microblepharon exfoliation (BlephEx™) on bacterial load and meibomian gland function. These randomized controlled trials demonstrated that microblepharon exfoliation significantly reduces the number of bacteria on the eyelid margin and improves meibomian gland secretion quality. However, it should be noted that these studies were not double-blinded and focused primarily on symptomatic contact lens wearers rather than non-contact lens patients with chronic blepharitis, as in our study. The specific population difference could account for some variability in outcomes, as the challenges of managing chronic blepharitis in a contact-lens-less population may differ from those of contact lens-related discomfort. Additionally, the lack of blinding in the Siddireddy studies may have introduced a placebo effect, as the mechanical stimulation during BlephEx™ treatment differs from that in the control treatment. This could have contributed to the positive outcomes reported in these studies. In contrast, our study was double-masked, which minimizes this type of bias and allows for a more reliable comparison of the treatment effect. This distinction may partially explain why our results did not show a statistically significant difference between BlephEx™ and sham treatment, especially in patients without contact lens wear. Additionally, while BlephEx™ may reduce bacterial load, this may not directly translate into sustained clinical improvements in symptoms for all patients with chronic blepharitis.

In relation to Demodex follicularis colonization of the eyelids, Epstein et al. [25] published a study in 2020 that demonstrated statistically significant reductions in Demodex when various substances were combined with the BlephEx™ treatment in a population of 46 patients. However, the authors noted that the clinical significance of these findings was unclear because there was no correlation between the Demodex levels and OSDI score. Thus, a reduction in Demodex might not result in a decrease in blepharitis-related complaints.

Two randomized controlled studies compared the effect of BlephEx™ with that of other treatment options. Mohammad-Rabei et al. [26] compared treatment with the BlephEx™ device in combination with tea tree oil (2% shampoo) via a sham BlephEx™ procedure (comparable to ours) combined with the same product. The authors reported a statistically significant superiority of the BlephEx™ group in terms of reducing the OSDI score and number of Demodex mites. In contrast, Murphy et al. [27] found no difference in efficacy in terms of symptom reduction or Demodex mite counts when comparing two different lid margin care products without BlephEx™ application to a group that underwent in-house lid scrubing with the BlephEx™ device followed by nightly lid scrubs. Thus, the study evidence remains sparse and contradictory.

Another recently published prospective, randomized clinical trial [28] also investigated the effect of BlephEx™ treatment on chalazion development and revealed a significantly greater resolution rate of chalazion in a group treated with BlephEx™ and lid margin hygiene (*n* = 23) than in the lid hygiene group alone (*p* = 0.007). Although these are promising data related to chalazion resolution, there is limited inference of the transferability of BlephEx™ to blepharitis without chalazion.

The most frequent side effect in our study was mild eye irritation immediately after treatment, which occurred in both groups roughly equally frequently (three patients in the BlephEx™ group and two patients in the sham group). Both the improvement in the Efron grading scale score in the sham treatment group and the adverse events, which were comparable to those in the treatment group, suggest a notable placebo effect.

In our study, the OSDI at inclusion was significantly greater in the Sham group than in the BlephEx™ group despite randomization; however, since the difference from baseline was calculated, no pronounced bias should have arisen. The limitations of this study could be the single treatment and the short follow-up period of four weeks. However, considering the low effect sizes of the OSDI, BUT, Schirmer and Efron grading Scale, a significant difference between the groups seems unlikely even after a longer observation period.

Another limitation is that the study did not assess variables such as microorganism load, lipase activity, collarette cure, and meibomian gland secretion quantity, which could provide additional academic insights. Instead, the focus was on the OSDI score, which directly reflects the patient's subjective experience and disease burden. However, as the OSDI is a key indicator of clinical effectiveness, its relevance to patient outcomes makes it one of the most critical parameters in evaluating treatment success.

In conclusion, this study represents the first randomized double-blind controlled trial comparing BlephEx™ treatment to a sham intervention without the use of additional products containing tea tree oil. Furthermore, compared to other studies, a broad range of subjective and objective blepharitis parameters was assessed, allowing for a more nuanced evaluation. This finding revealed that the BlephEx™ treatment did not demonstrate statistically significant benefits over sham treatment for managing blepharitis in this study.

## Data Availability

The datasets generated and analyzed during the current study are not publicly available but are available from the corresponding author upon reasonable request.

## Abbreviations

CI: Confidence Interval
ETDRS: Early Treatment of Diabetic Retinopathy Study
IQR: Interquartile range
OD: Right Eye
OS: Left Eye
OSDI: Ocular Surface Disease Index
TBUT: Tear Break-Up Time
VA: Visual Acuity
